# Supplementary material for: Structure of the herpes simplex virus type 2 C-capsid with capsid-vertex-specific component
Source: Nat Commun. 2018 Sep 10;9:3668. doi: 10.1038/s41467-018-06078-4 (PMC6131487; doi:10.1038/s41467-018-06078-4)
Supplement: Supplementary file 1 — Supplementary Information [file 41467_2018_6078_MOESM1_ESM.pdf]

Supplementary Information

Structure of the Herpes simplex virus type 2 C-capsid with capsid-vertex-specific component

J. Wang, S. Yuan, D. Zhu et al.

List of Content

Supplementary Figures 1-8

Supplementary Table 1

Supplementary References

Supplementary Figures

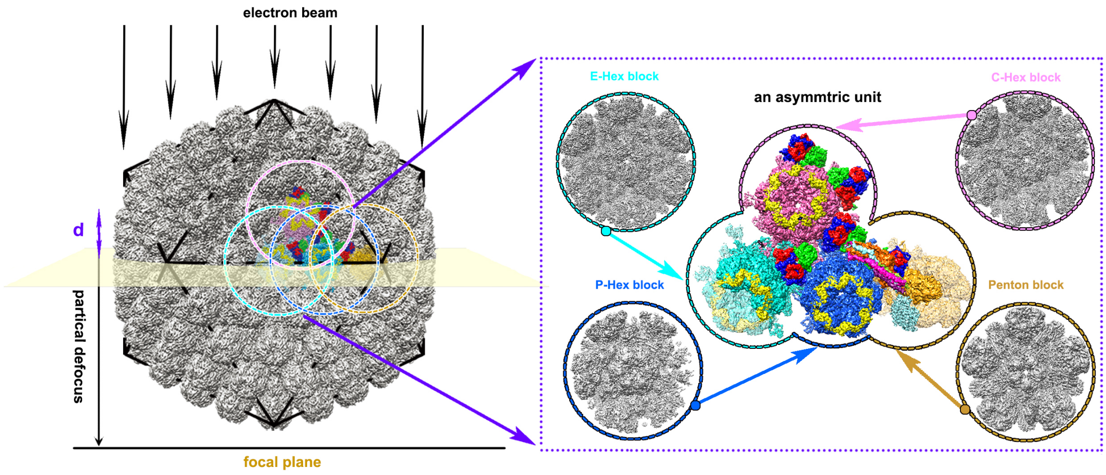

Supplementary Figure 1

Block-based reconstruction method.
Insets show how block-based reconstruction works. A density map was divided into four blocks with the center locating in Penton, P-Hex, C-Hex and E-hex, which are circled with yellow, blue, magenta and cyan dashes, respectively. The distance between the center of mass of the model and the focal plane of objective lens along the Z axis is the particle defocus. Each block has its own local mean focus, which is the sum of the particle defocus values with the distance from the center of the block to the center of the model along the Z axis.

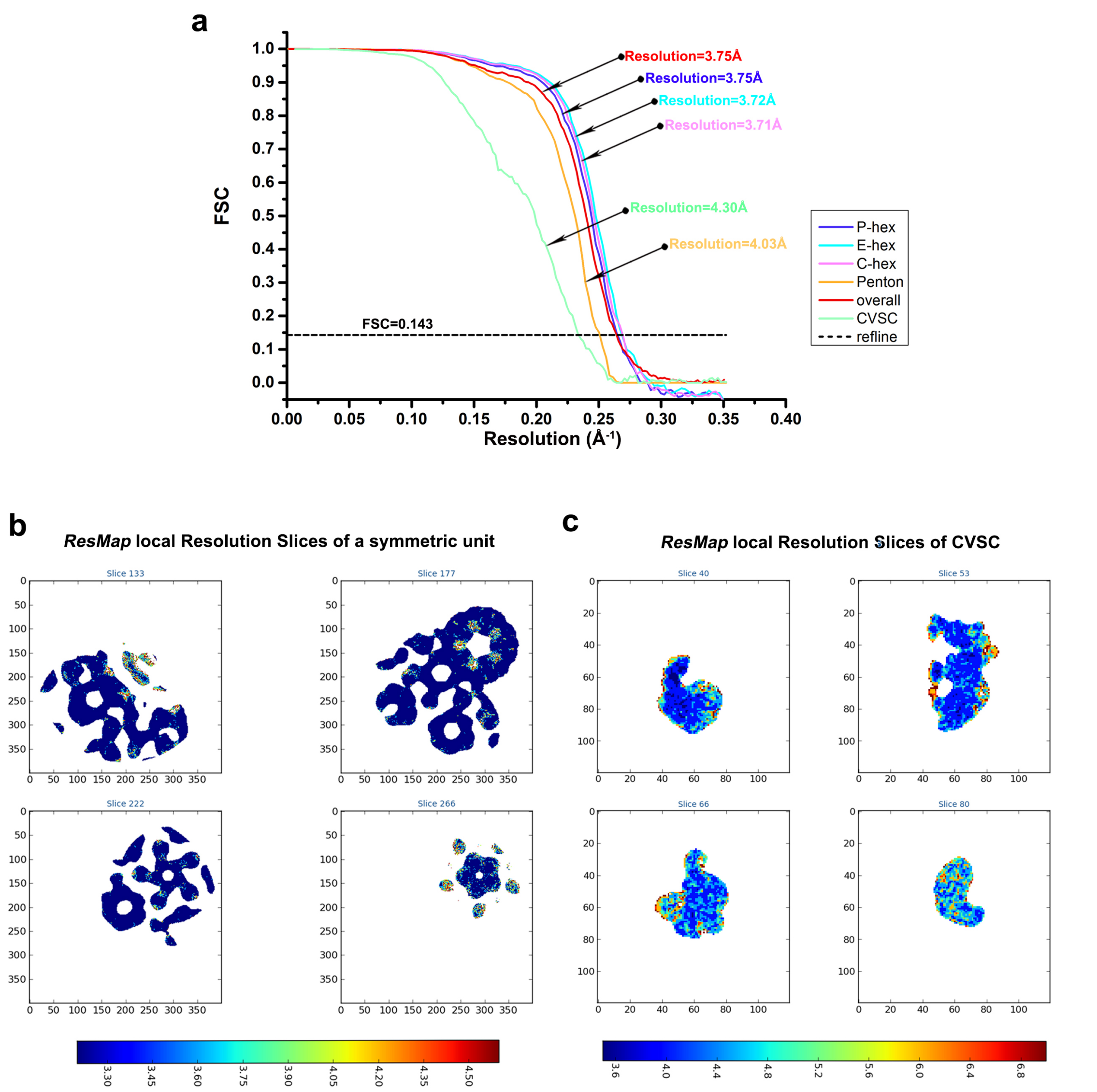

Supplementary Figure 2

Resolution assessment of the cryo-EM map.

(a) The calculated gold-standard FSC curve. Based on the FSC=0.143 criterion, the resolution of the four blocks, CVSC and an asymmetric unit maps are shown. (b) and (c) Local resolution assessments of an asymmetric unit and the CVSC. Local resolution heat maps of density slices through an asymmetric unit and the CVSC are rendered using ResMap[1]. The red to blue color scheme corresponds to regions of relative low to high resolution.

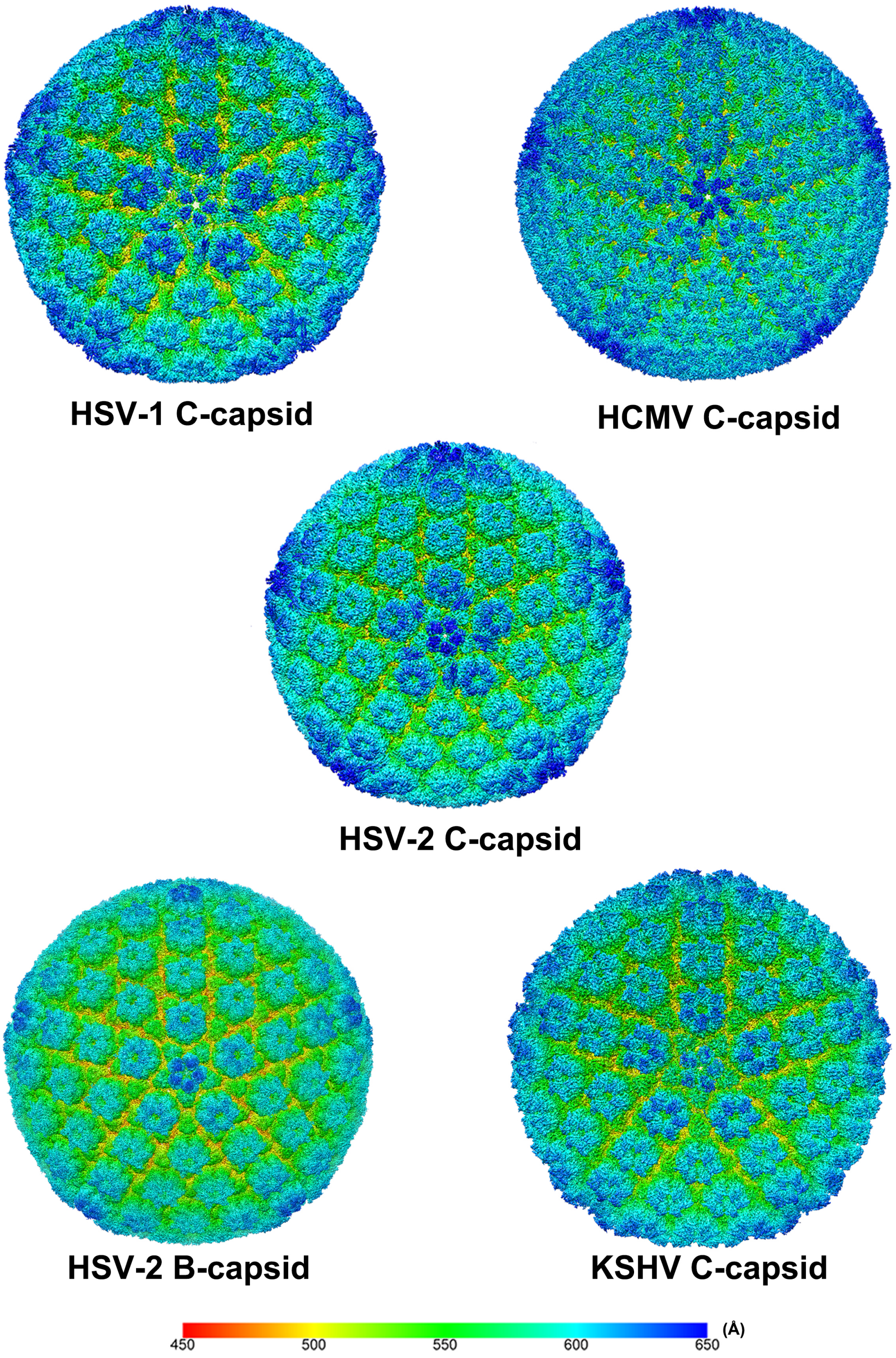

Supplementary Figure 3

Radially colored reconstructions of HSV-2 B- and C-capsids, HSV-1 and HCMV and KSHV virions viewed along a fivefold axis.

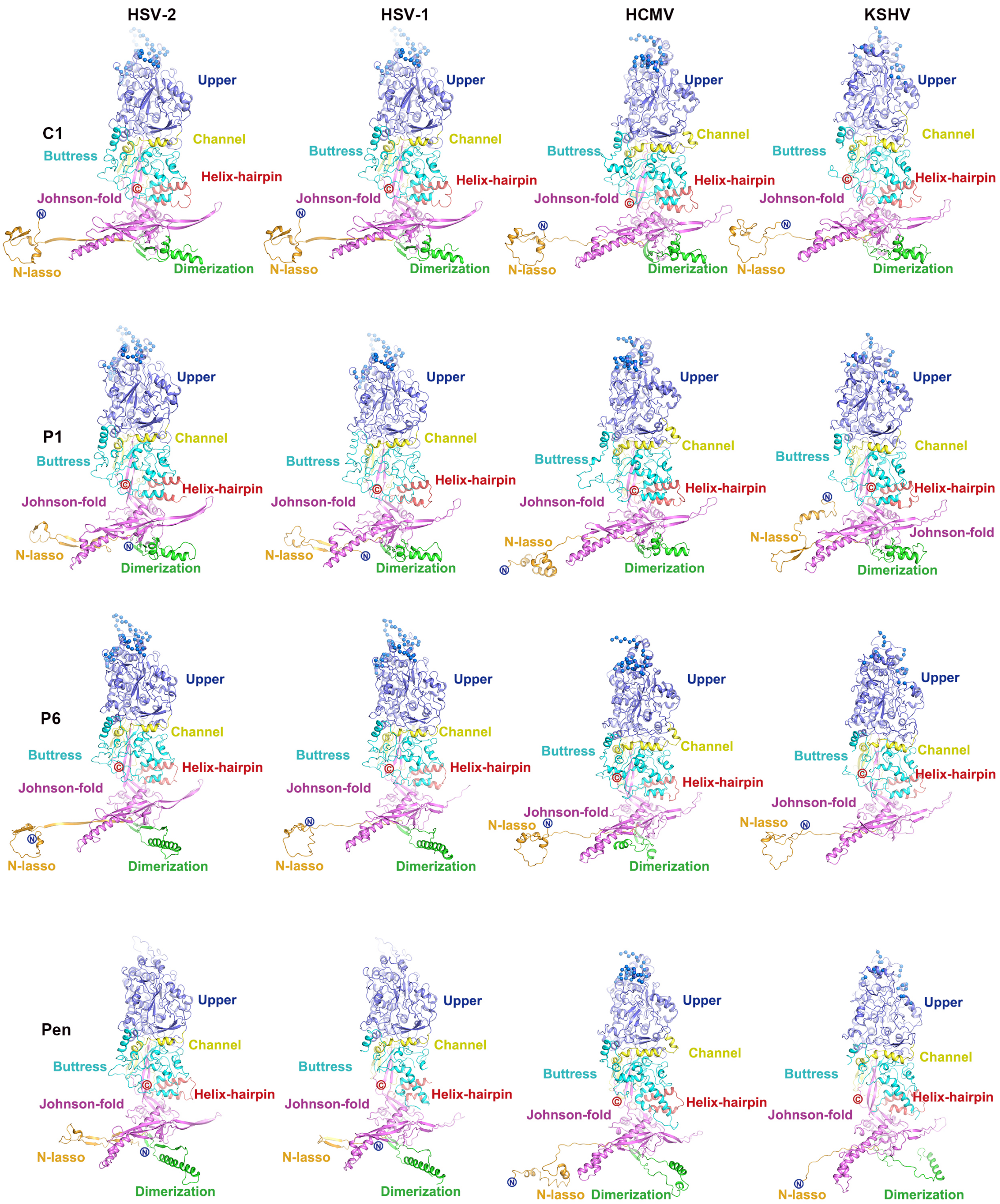

Supplementary Figure 4

Structural comparisons of C1, P1, P6, Pen1 from HSV-2, HSV-1, HCMV and KSHV, which are colored and labeled by domain.

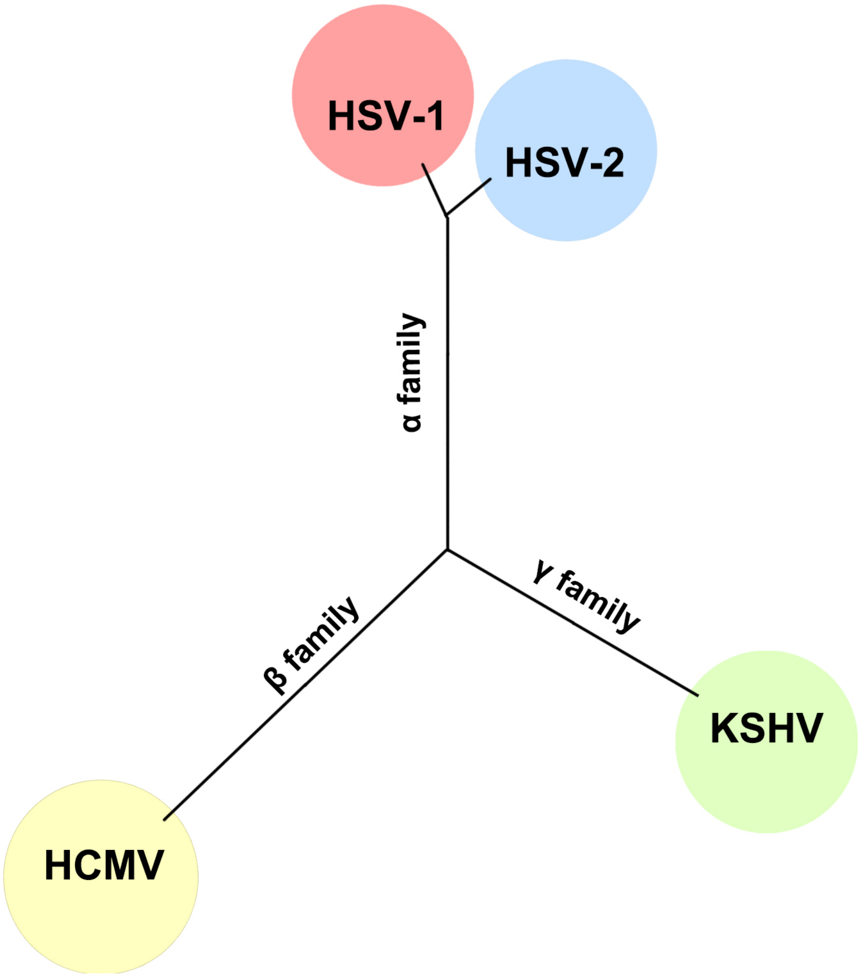

Supplementary Figure 5

Structure-based phylogenetic tree of the asymmetric unit from HSV-1, HSV-2, HCMV and KSHV. The length of the line reflects distance of genetic relationship.

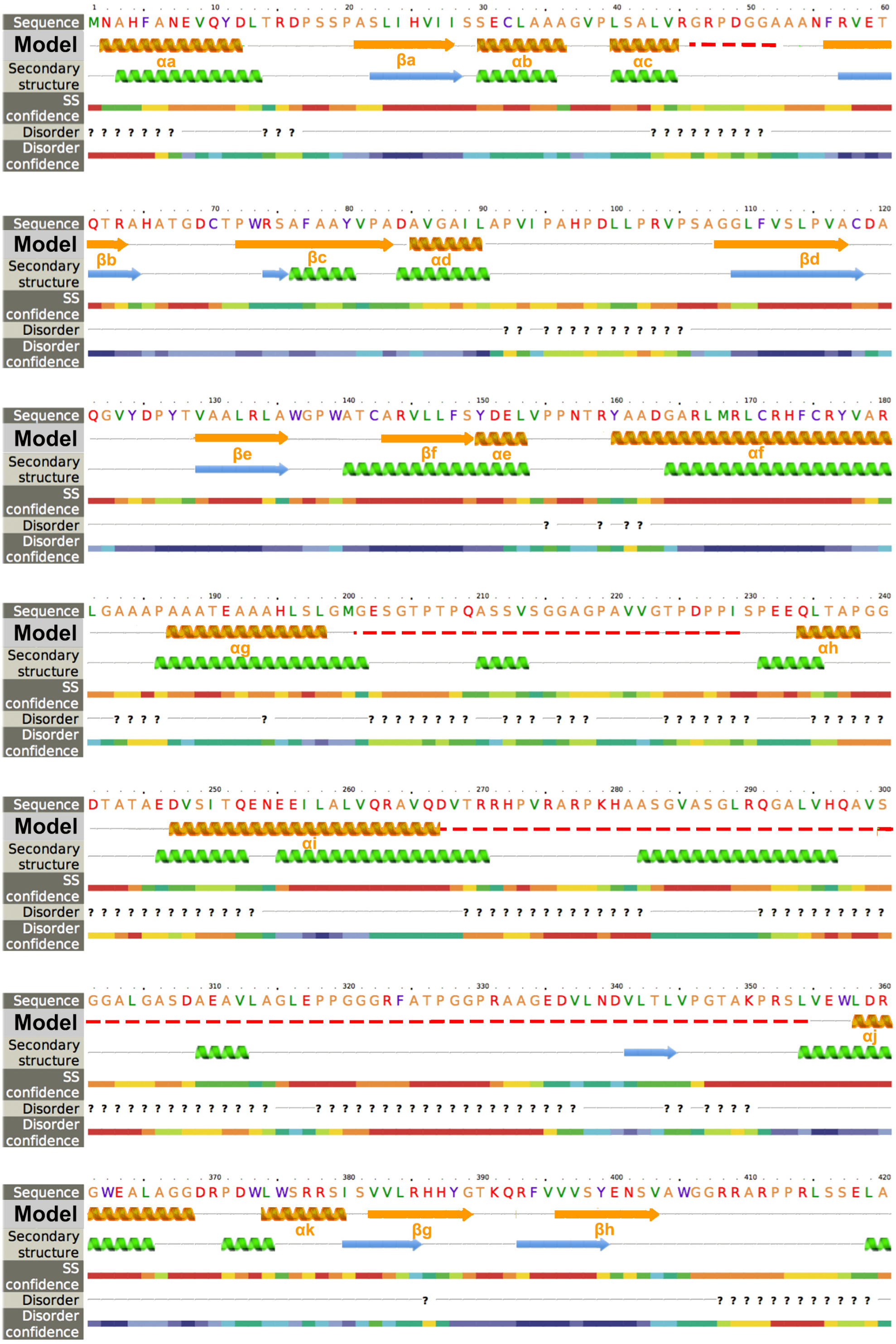

Sequence
Model
Secondary structure
SS confidence
Disorder
Disorder confidence
MNAHFANEVQYDLTRDPSSPASLIHVIISSECLAAAGVPLSALVRGRPDGGAAANFRVET
αa βa αb αc
QTRAHATGDCTPWRSAFAAYVPADAVGAILAPVIPAHPDLLPRVPSAGGLFVSLPVACDA
βb βc αd βd
QGVYDPYTVAALRLAWGPWATCARVLLFSYDELVPPNTRYAADGARLMRLCRHFCRYVAR
βe βf αe αf
LGAAAPAAATEAAAHLSLGMGESGTPTPQASSVSGGAGPAVVGTPDPPISPEEQLTAPGG
αg αh
DTATAEDVSITQENEEILALVQRAVQDVTRRHPVRARPKHAASGVASGLRQGALVHQAVS
αi
GGALGASDAEAVLAGLEPPGGGRFATPGGPRAAGEDVLNDVLTLVPGTAKPRSLVEWLDR
αj
GWEALAGGDRPDWLWSRRSISVVLRHHYGTKQRFVVVSYENSVAWGGRRARPPRLSSELA
αk βg βh

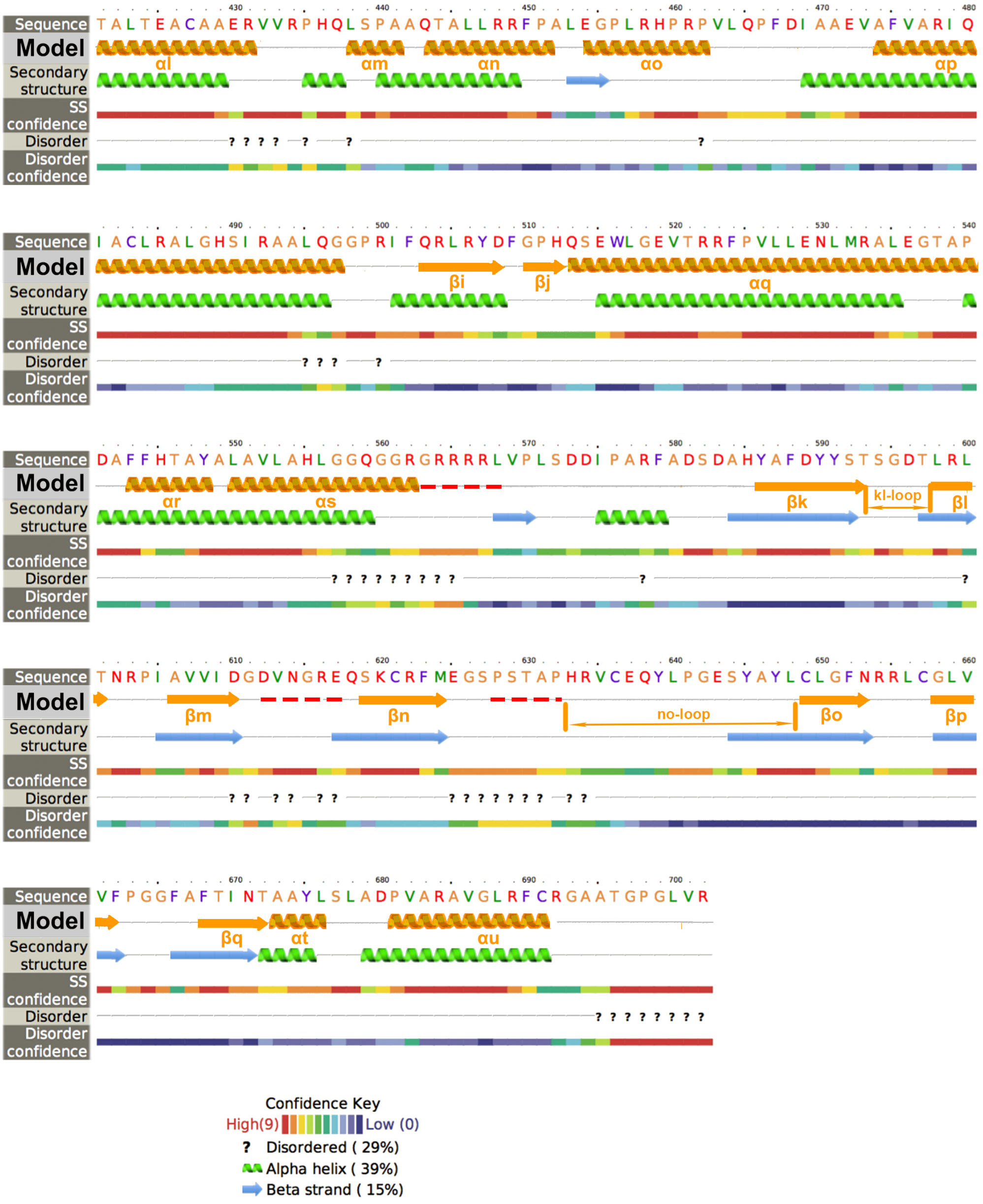

Supplementary Figure 6

Model validation of UL17. PHYRE2 Protein Fold Recognition Server[2] was used to predict the secondary structural elements of UL17. The model of UL17 colored as orange is showed in insets.

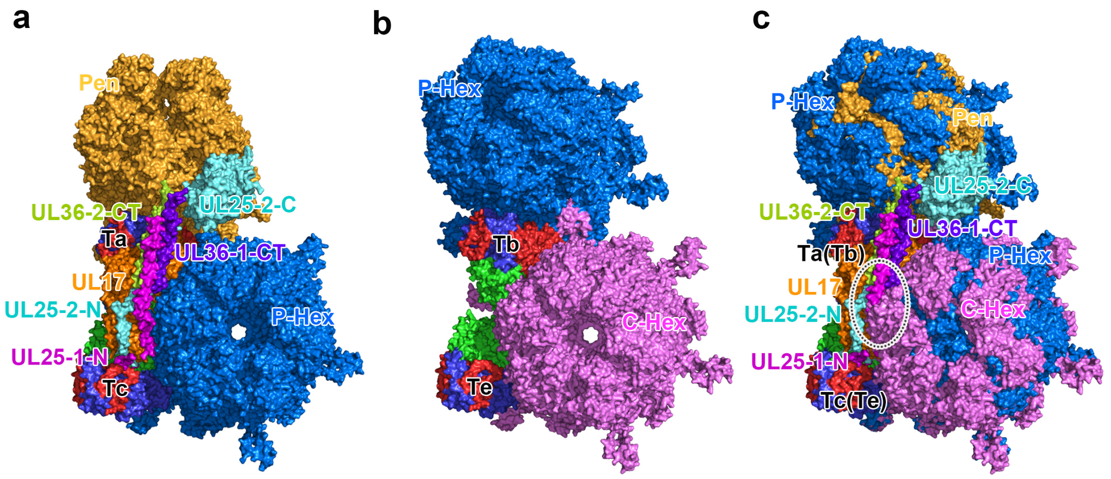

Supplementary Figure 7

The CVSC molecule binds only to a specific triplex pair (Ta-Tc) adjacent to vertices. (a) Surface representation of the CVSC and its contacting microenvironment. (b) Surface representation of the triplex pair (Tb-Te) with the same arrangement as that of Ta-Tc and its surrounding hexons. (c) Superimposition of (a) and (b) based on the triplex pairs suggests that differences in the relative orientation of the specific pair (Ta-Tc) with its contacting capsomers exclude the binding of the CVSC to the triplex pair (Tb-Te) via steric hindrance. The color scheme is same as in Fig. 1c.

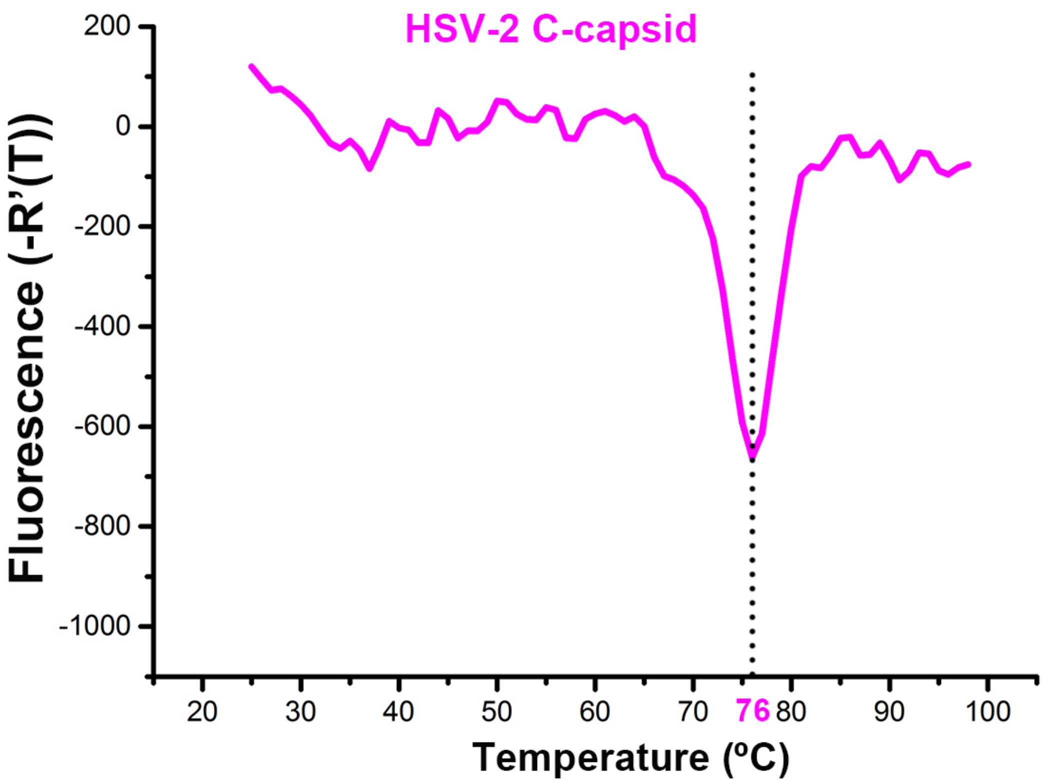

Supplementary Figure 8

The stabilities of HSV-2 C-capsids were determined by thermofluor assay using the dyes SYTO-Green and SYTO9 to detect DNA and RNA exposures, respectively. The first derivatives (−R‘‘(T)) of the raw fluorescence traces of HSV-2 C-capsids (fluorescence was measured in triplicate) is shown.

Supplementary Tables

Supplementary Table 1

Cryo-EM data collection, refinement and validation statistics

	HSV-2 C-capsid (EMDB-6976) (PDB 5ZZ8)
**Data collection and processing**	
Magnification	59000
Voltage (kV)	300
Electron exposure (e–/Å^2)	25
Defocus range (μm)	0.8-2.3
Pixel size (Å)	1.41
Symmetry imposed	C1
Initial particle images (no.)	64659
Final particle images (no.)	56901
Map resolution (Å)	3.75
FSC threshold	0.143
Map resolution range (Å)	3.3-4.5
**Refinement**	
Initial model used (PDB code)	5ZAP
Model resolution (Å)	3.1
FSC threshold	0.143
Model resolution range (Å)	3.0-4.2
Map sharpening *B* factor (Å^2)	-150
Model composition	
Non-hydrogen atoms	216030
Protein residues	28753
Ligands	0
B factors (Å^2)	
Protein	30
Ligand	
R.m.s. deviations	
Bond lengths (Å)	0.010
Bond angles (°)	1.034
Validation	
MolProbity score	2.24
Clashscore	18
Poor rotamers (%)	0.1%
Ramachandran plot	
Favored (%)	94.26%
Allowed (%)	5.17%
Disallowed (%)	0.57%

Supplementary References

1. Kucukelbir, A., Sigworth, F.J. & Tagare, H.D. Quantifying the local resolution of cryo-EM density maps. *Nat Methods* **11**, 63-5 (2014).
2. Kelley, L.A., Mezulis, S., Yates, C.M., Wass, M.N. & Sternberg, M.J. The Phyre2 web portal for protein modeling, prediction and analysis. *Nat Protoc* **10**, 845-58 (2015).
